# The impact of immunosuppression on the mortality and hospitalization of Monkeypox: a systematic review and meta-analysis of the 2022 outbreak

**DOI:** 10.1186/s12985-024-02392-0

**Published:** 2024-06-05

**Authors:** Ahmed Azzam, Heba Khaled, Haitham Salem, Ameer Ahmed, Amira M. Heniedy, Hassan Samy Hassan, Ahmed Hassan, Taghrid S. El-Mahdy

**Affiliations:** 1 Department of Microbiology and Immunology, Faculty of Pharmacy, Helwan University, Cairo, Egypt; 2 Department of Biochemistry, Faculty of Pharmacy, Cairo University, Cairo, Egypt; 3 Faculty of Medicine, Ain Shams University, Cairo, Egypt; 4 Faculty of Medicine, Minia University, Minya, Egypt; 5 Department of Epidemiology, El-Beheira Veterinary Administration, Egyptian Ministry of Agriculture and Land Reclamation, El-Beheira, Egypt; 6 Faculty of Pharmacy, Tanta University, Tanta, Egypt; 7 Dermatology resident physician, Qeft Teaching Hospital, Qena, Egypt; 8 Department of Microbiology and Immunology, Faculty of Pharmacy, Modern University for Technology and Information (MTI), Cairo, Egypt

**Keywords:** Monkeypox, Mpox, HIV, Hospitalization, Mortality, Immune reconstitution inflammatory syndrome, Meta-analysis

## Abstract

**Background:**

Limited data is available regarding the severity and mortality of Mpox in individuals with immunocompromised conditions. Therefore, we performed this meta-analysis to understand the impact of HIV- or non-HIV-associated immunosuppression on the severity of Mpox requiring hospitalization and mortality.

**Methods:**

A thorough literature search was performed from 2022 up to January 2024. The results were presented as odds ratios (ORs). We only included patients who required hospitalization for severity rather than isolation.

**Results:**

A total of 34 studies were included in this analysis. Our analysis did not find a significant difference in the hospitalization risk between HIV-positive individuals and those who were HIV-negative (OR = 1.03; *P* = 0.85; 7 studies; CD4 count of fewer than 200 cells/µL was less than 0.5% across all studies). Patients with a CD4 count lower than 200 cells/µL or an unsuppressed RNA viral load (> 200 copies/ml) had a significantly higher hospitalization risk (OR = 5.3, *P* < 0.001) and (OR = 3, *P* < 0.001), respectively. Most of the reported deaths were reported in patients with HIV with CD4 counts below 200 cells/µL, with some fatal cases occurring in non-HIV immunosuppressed patients, particularly organ transplant recipients. Based on the autopsy findings, Mpox was confirmed in multiple organs, particularly the digestive tract, lung, and testes. Furthermore, some studies documented cases of death that were suspected to be related to hemophagocytic lymphohistiocytosis (HLH) and immune reconstitution inflammatory syndrome (IRIS). Most of the death reports showed concomitant non-Mpox infections at the time of hospitalization and death

**Conclusions:**

Our finding shows that Mpox acts as an opportunistic pathogen in immunocompromised individuals. These individuals should be prioritized for early care and closely monitored for signs of deteriorating clinical conditions. Clinical manifestations and autopsy findings strongly suggest Mpox dissemination to multiple organs, particularly the digestive tract, and lungs. However, the presence of concomitant non-Mpox infections complicates the assessment of the attribution of Mpox to death. Caution should be exercised when interpreting data suggesting poorer outcomes in individuals with non-HIV immunosuppression, as current evidence is scarce and further research is needed.

**Supplementary Information:**

The online version contains supplementary material available at 10.1186/s12985-024-02392-0.

## Introduction

Mpox (previously known as Monkeypox) is a neglected, reemerging viral infection that is endemic in East Central and West Africa [1]. From 2022 onward, Mpox has evolved into a global epidemic, impacting numerous countries beyond the African continent, where Mpox had not been reported before. In July 2022, the World Health Organization (WHO) designated the increasing Mpox outbreak as a Public Health Emergency of International Concern (PHEIC) [2]. However, in May 2023, WHO declared the end of the Mpox emergency as it noted a significant decline in the number of reported cases compared to the previous reporting period and no changes in the severity or clinical manifestation of the disease [3].

Mpox is a zoonotic disease caused by an Orthopoxvirus. The virus is categorized into two distinct clades. Clade I, originating from Central Africa, exhibits a mortality rate exceeding 10% and demonstrates higher transmissibility [4–6]. Clade II, on the other hand, has a mortality rate of less than 1% and is subdivided into clades IIa (prevalent in West Africa) and IIb (identified in the ongoing Nigerian outbreak of 2017 and the current outbreak in 2022) [6]. A previous meta-analysis revealed that about 40.32% of Mpox cases had HIV co-infection [7]. The vast majority of cases occur in men (91.44%) especially Men Who Have Sex With Men (MSM) [7].

During the 2022 Mpox outbreak, the hospitalization rate is estimated to be 5.8% [8], while mortality is relatively uncommon, with approximately 1.3 to 1.2 Mpox-associated deaths per 1,000 cases [9]. Emerging studies indicate that individuals with uncontrolled HIV have an increased risk of hospitalization and potentially higher mortality rates if they contract Mpox compared to individuals without HIV. This heightened vulnerability in people with advanced HIV (CD4 counts < 200 cells/µL) can be explained by two key factors. First, the severe CD4 + T-cell depletion could impair the body’s ability to control Mpox viral replication, leading to viral dissemination and potential organ failure [10]. Second, it may be related to immune dysregulation mechanisms such as lymphohistiocytosis (HLH) [11–13] or immune reconstitution inflammatory syndrome (IRIS) [12, 14–19]. However, the evidence is limited due to the small number of patients with uncontrolled HIV and the overall low hospitalization and mortality rates in the current outbreak. In addition, our knowledge of the impact of non-HIV immunocompromised conditions on Mpox severity and the mechanisms by which Mpox can cause severe illness is limited. Therefore, it is necessary to synthesize data from high-quality studies to overcome the limitations of individual studies. The findings from this meta-analysis would help identify specific populations that should be prioritized for early antiviral therapy, vaccination, and other preventive measures.

## Methods

### Search strategy

A thorough literature search was performed from January 1st, 2022, up to January 15th, 2024, using the following databases: MEDLINE [PubMed], Scopus, Google Scholar, and Web of Science. The search strategy is shown in Table S1. Additionally, the reference lists of the included studies were scanned to ensure a thorough representation of the existing literature. This study was conducted according to the Preferred Reporting Items for Systematic Reviews and Meta-Analyses (PRISMA) statement [20]. Table S2 shows the 27 items of the PRSIMA checklist.

### Eligibility criteria

#### Inclusion criteria

The study was included if it met one of the following criteria: (1) studies reporting hospitalization of Mpox cases in patients with non-HIV immunocompromised conditions in comparison to immunocompetent controls; (2) studies comparing hospitalization rates between HIV-positive patients and HIV-negative patients, with clear data on CD4 cell counts or RNA viral load of the HIV-positive individuals; (3) studies examining hospitalization rates within subgroups of viral load or CD4 cell counts in HIV-positive individuals; and (4) studies reporting Mpox-related deaths and providing clear data on the Immunosuppression source of the patients. We only included studies that hospitalized patients based on severity rather than infection control or isolation to provide a precise link between hospitalization and severity.

#### Exclusion criteria

We excluded studies that didn’t report or had substantial missing data regarding CD4 cell counts or RNA viral load. Two independent reviewers screened eligible articles from the electronic search outputs based on the inclusion and exclusion criteria. Disagreements were solved by discussion and consensus between the two reviewers.

#### Data extraction

Two independent reviewers extracted the necessary information from the included articles. The extracted data was then cross-checked by another reviewer to ensure reliability and accuracy. For studies that compare the hospitalization of Mpox between HIV-positive and HIV-negative patients, the following information was extracted: last name of the first author, publication year, country, study design, number of centers, number of confirmed Mpox cases, age category, data on HIV immune status (CD4 counts, HIV RNA), percentage of hospitalization in HIV-positive patients and HIV-negative patients. For studies that stratify the hospitalization rate in HIV patients by CD4 count or RNA load, the following information was extracted: last name of the first author, publication year, country, study design, number of centers, age category, and study main finding. For studies that report deaths, the following items were extracted: last name of the first author, country, number of deaths, HIV status, immunosuppression source, deterioration after initiation of ART, cause of death, non-Mpox concomitant infection at the time of hospitalization and death, and evidence of Mpox dissemination based on autopsy findings.

### Quality assessment

We adopted the New Castle-Ottawa quality assessment scale for cohort studies to assess the quality of the included studies [21]. The checklist items are presented in Tables S3 and S4.

### Data synthesis

The results were presented as odds ratio (OR) with a 95% confidence interval (CI) calculated using the random-effects model. Heterogeneity between the studies was assessed using I-squared %. Sensitivity analysis was performed utilizing the leave-one-out technique to examine the robustness of the results. Publication bias testing was not conducted if the number of included studies was less than ten. All statistical analyses were performed using Comprehensive Meta-Analysis version 3.0 (Biostat, Englewood, NJ, USA).

## Results

### Characterization of the included studies

**Fig. 1:**
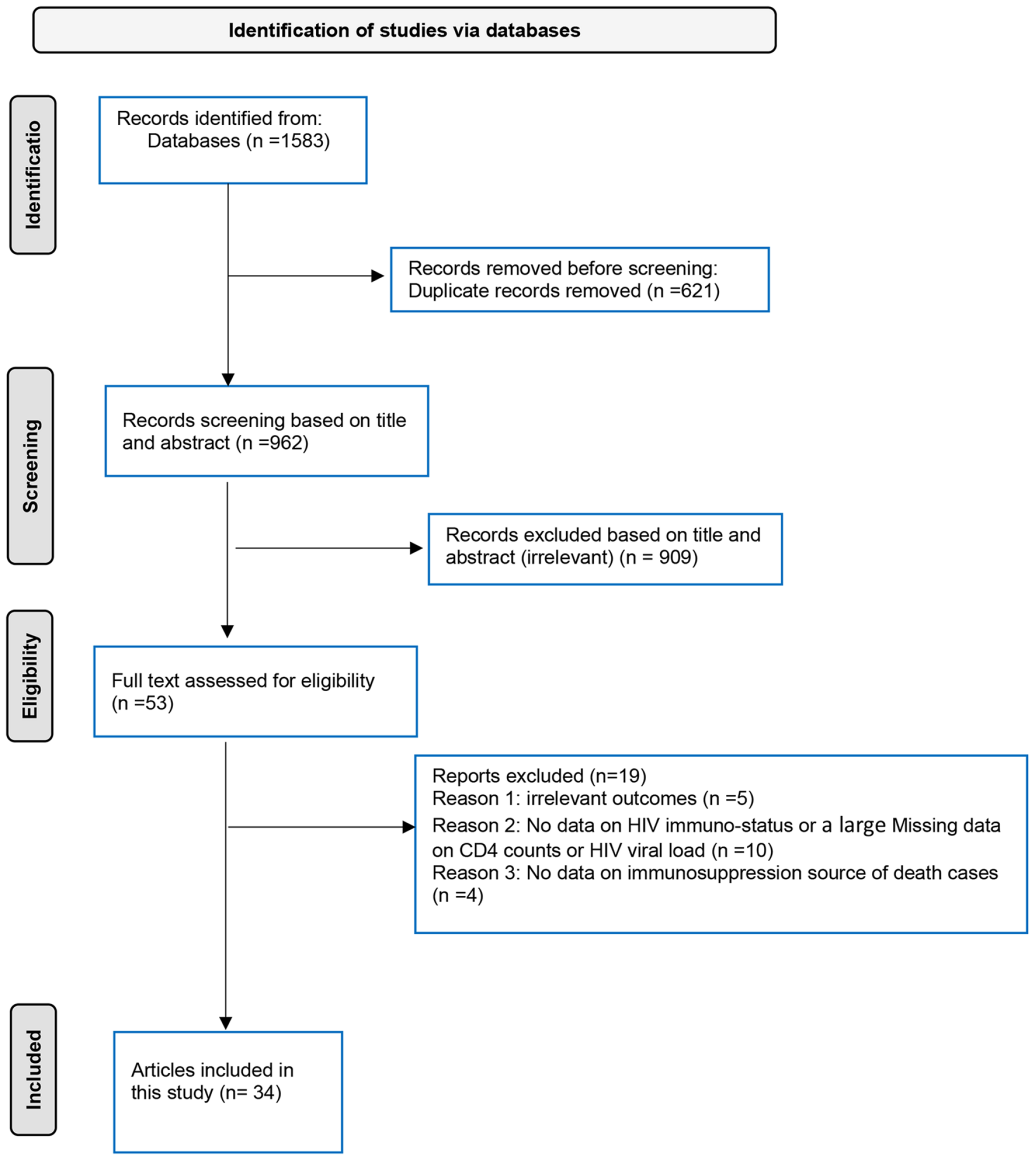
PRISMA flow diagram depicting the selection of the included articles

A total of 34 studies were included in the analysis [11–19, 22–46] as shown in Fig. 1. Ten studies compared the hospitalization of Mpox between HIV-positive and HIV-negative patients [22–30, 46], as shown in Table 1. Only one report of hospitalization risk for patients with non-HIV immunocompromised conditions relative to HIV-negative and immunocompetent [44]. Five studies stratified Mpox outcomes based on CD4 and or RNA viral load [15, 23, 25, 26, 40] (Table 2). Furthermore, 24 studies provided data on deaths among Mpox cases and included information on Immunosuppression source [11–19, 25, 31–43, 45], as shown in Table 3.

**Table 1:** The characteristics of the included studies that compare the hospitalization of Mpox between HIV-positive and HIV-negative patients

Author, Publication year	Country	Study design	# Centers	# Confirmed MPXV infections	Age category	CD4 count cells/µL	HIV RNA undetectable values or less than 200 copies/ml %	% of hospitalization in HIV-positive, (event/total)	% of hospitalization in HIV-negative, (event/total)
A: Studies with a low proportion of low CD4									
Angelo 2023 [24]	Multi-national	Cross-sectional	Multicenter	226	Adult	1% < 200	92	6.5 (6/92)	7.5 (10/134)
Caria 2022 [27]	Multi-national	Cross-sectional	Multicenter	41	Adult	0%<200	88	8 (2/25)	12.5(2/16)
Hoffmann 2023 [22]	Germany	Retrospective cohort	Multicenter	546	Adult	0% <185, 2.9% < 350	95.8	2.7(7/256)	5.2(3/58)
Martín-Iguacel 2023 [23]	Spain	Prospective cohort	Multicenter	2122	Adult	0% < 200	87.6 *	1.9(13/661)	1.7(22/1280)
Philpott 2023 [26]	USA	Retrospective cohort	Unicenter	1921	Adult	0% < 200	100	4.6(52/1124)	4.6(37/797)
Pilkington 2023 [46]	United Kingdom	Retrospective cohort	Unicenter	150	Adult	5% < 200	92.4% *	18.9 (11/58)	9.09 (8/88)
Silva 2023 [25]	Brazil	Prospective cohort	Unicenter	418	Adult	0% <200	NA	8.7 (16/183)	9.7(19/196)
B: studies with a high proportion of low CD4									
Brosnan 2023 [30]	USA	Cross-sectional	Multicenter	118	Adult	14% <200	49	26.7 (19/71)	12.7(6/47)
Estevez 2023 [28]	Spain	Retrospective cohort	Unicenter	100	Adult	59.3% < 350	93.1	7(5/71)	3.57 (1/28)
Chastain 2023 [29]	USA	Retrospective cohort	Multicenter	322	Adult	587 ± 371^β^	14,734 ± 20 640^β^	10.8(10/92)	4.34 (4/92)

*Note* * HIV RNA < 200 copies/ml, β mean ± standard deviation

**Table 2:** Studies examining the risk of hospitalization in non-HIV immunocompromised individuals or HIV-positive individuals based on CD4 count or RNA viral load

Author, Publication year	Country	Study design	# Centers	Age category	Main findings
a: Studies examining the risk of hospitalization in HIV-positive individuals based on CD4 count or RNA viral load					
Philpott, 2023 [26]	USA	Retrospective study	Multicenter	Adult	Compared with patients without HIV* 1-PWH with a CD4 count < 350 cells/mm3 and (a) unsuppressed VL RR = 3.6 (95% CI, 2.0–6.4),(b) Suppressed VL RR = 2.3 (95% CI, 1.2–4.4) 2- PWH with a CD4 count ≥ 350 cells/mm3, (a) suppressed VL RR = 0.9 (95% CI, 0.5–1.5),(b) unsuppressed VL RR = 1.5 (95% CI, 0.6–3.5).
Martín-Iguacel, 2023 [23]	Spain	Prospective study	Multicenter	Adult	PWH who have a CD4 cell count below 200 cells/µL and an HIV-RNA level equal to or greater than 50 copies/mL experienced a significantly higher rate of hospitalization.
Mitjà, 2023 [15]	Multinational	Retrospective cohort	Multicenter	Adult	Declining CD4 cell counts and increasing VL were associated with higher rates of hospitalization. No deaths among PWH and CD4 levels greater than 200 cells/µL. The risk of death increased gradually for PWH with CD4 levels less than 200.
Silva, 2023 [25]	Brazil	Prospective cohort study	Unicenter	Adult	The severity of Mpox was found to be associated with poor outcomes in individuals with HIV, particularly those with low CD4 + cell counts.
Aldred, 2023 [40]	USA	Retrospective cohort	Multicenter	Adult	Hospitalization was highest in PWH with HIV VL > 200 copies/mL at 41.1%, then 13.2% in PWH with HIV VL ≤ 200 copies/mL, and 9.1% in PWoH.
b: Studies examining the risk of hospitalization in non-HIV immunocompromised individuals					
Laurenson-Schafer, 2023 [44]	Multinational	Retrospective surveillance	Multicenter	Mixed	Cases who were HIV-negative and immunocompromised exhibited a significantly higher risk (adjusted OR = 3.47, 95% CI [1.84 to 6.54], *P <* 0.001) compared to cases who were HIV-negative and immunocompetent.

*Note* * based on multivariate analysis, PWoH: People without HIV, PWH People with HIV, VL: Viral load, RR: risk ratio

**Table 3:** Characteristics of death cases of Mpox

Author	Country	# death, HIV status	Immunosuppression sources	Deterioration after initiation of ART*	Non-Mpox concomitant infections^α^
Garneau, 2023 [14]	USA	2 (+)	Uncontrolled HIV, (CD4 + less than 50 cells/µl and HIV VL of more than 2000 copies/mL)	Yes, both	-
Aldred, 2023 [40]	USA	1 (+)	Uncontrolled HIV, VL > 200 copies /mL, and CD4 < 100 cells/µL)	-	-
Mitjà, 2023 [15]	Multinational	27 (+)	Uncontrolled HIV, (CD4 counts of less than 200 cells/µL)	Yes, (21 were suspected of having IRIS)	Yes
Silva, 2023 [25]	Brazil	2	Uncontrolled HIV, (CD4 + less than 50 cells/µl)	-	-
Núñez, 2022 [34]	Mexico	1	Uncontrolled HIV, (CD4 + T cell count, 7 cells/µl)	-	Yes
Riser, 2023 [16]	USA	31 (+), 2(-)	31 of 33 had CD4 counts < 200 cells/µL and 2 non-HIV immunocompromised	Yes, 13 deaths were suspected with IRIS	-
Álvarez-Moreno, 2023 [33]	Colombia	2 (+)	Uncontrolled HIV in One patient (CD4 cell count of 33 cells/µL and a VL of 32,000). There is no data about the other.	-	Yes
Caria, 2023 [38]	Brazil	1(+)	Uncontrolled HIV, (CD4 + count of 173/µL)	-	Yes
Carrubba, 2023 [37]		2 (+)	Uncontrolled HIV, (CD4 + count of < 100 cells/µL) and VL > 40,000 copies per mL	-	Yes
Menezes, 2022 [36]	Brazil	1(+)	Uncontrolled HIV, (CD4 count was 5 cells/µL with undetectable VL)	-	-
Farias, 2023 [35]	Brazil	1(+)	Uncontrolled HIV, (CD4 + cell count < 50 cells/µL)	-	Yes
Rajme-López, 2023 [17]	Mexico	3(+)	Uncontrolled HIV, (The CD4 + cell count < 200 cells/µL and the unsuppressed RNA VL)	Yes, all of them are Suspected with IRIS	No
Warner, 2023 [18]	USA	1(+)	Uncontrolled HIV (the CD4 + cell count is 12 cells/µL with unsuppressed RNA VL)	Yes	Yes
Triana-González, 2023 [32]	Mexico	5 (4+) and (1-)	Uncontrolled HIV, (Four had a CD4 + cell count < 200 cells/µL and an unsuppressed VL), and one was non-HIV immunocompromised	-	Yes
Petti, 2023 [19]	USA	2 (+)	Uncontrolled HIV, (CD4 + cell count < 200 cells/µL and unsuppressed VL)	Yes, both patients were suspected with IRIS	Yes
Filippov, 2023 [31]	USA	1(+)	Uncontrolled HIV, (CD4 count was 25 cells/µL and VL was 678,000 copies/ml)	-	-
Alarcón 2023 [11]	USA	1(+)	Uncontrolled HIV, (CD4 + T-cell count, < 35 cells/µL)	-	Yes
Fuller 2023 [39]	USA	1(-)	kidney transplant recipient	-	Yes
Govind 2023 [12]	USA	3(+)	Uncontrolled HIV, (CD4 + cell counts < 100 cells/µL and VL > 100,000 copies/ml.)	Yes, 2 out of 3 were suspected with IRIS, with no data on the history of ART in the third case.	Yes
Ritter, 2024 [43]	USA	20 (+), 2(-)	Uncontrolled HIV (CD4 + cell counts < 150 cells/µL in 20 patients) and 2 immunocompromised patients with renal transplant recipients	-	Yes
Duarte-Neto, 2023 [13]	Brazil	2 (+)	Uncontrolled HIV (AIDS)	-	Yes
Mohammed, 2023 [42]	USA	1(+)	Uncontrolled HIV (AIDS)	-	Yes
SUN, 2023 [41]	-	1(+)	Uncontrolled HIV (AIDS)	-	Yes
Higgins, 2023 [45]	USA	1(-)	Renal transplant recipient	-	Yes

*Note* * The patient was suspected of having IRIS based on the findings of the included study, α: a detailed list of pathogens identified is present in Table S5, IRIS: immune reconstitution inflammatory syndrome, ART: Antiretroviral therapy, VL: viral load, (-): no data

### Hospitalization risk in controlled HIV-positive patients compared to HIV-negative patients

**Fig. 2:**
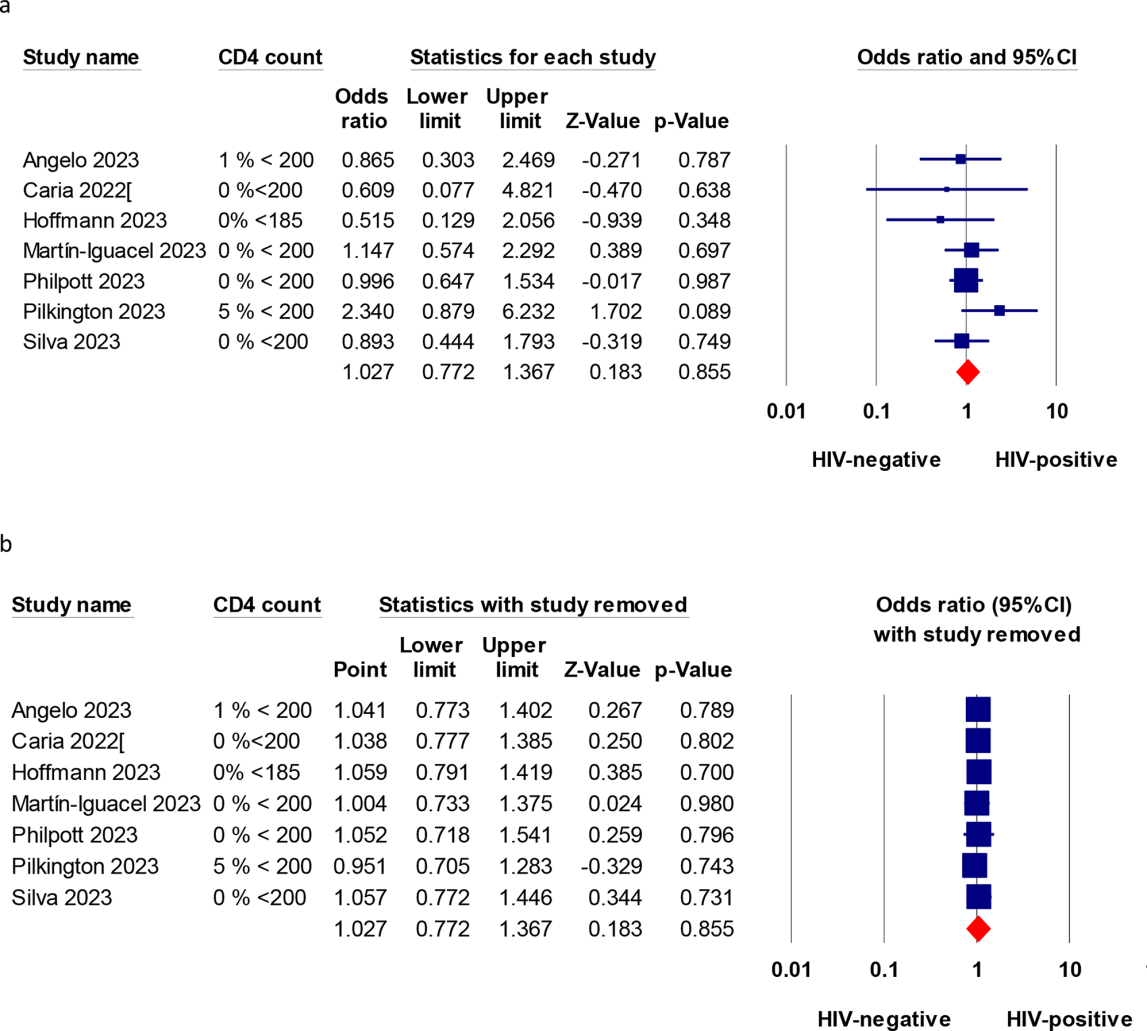
The pooled hospitalization risk among Mpox cases in people with controlled HIV infection relative to negative control. across all studies, the proportion of HIV patients with CD4 counts of less than 200 cells/µL did not exceed 0.5%. (**a**) the pooled hospitalization risk among HIV-positive patients compared with HIV-negative control; (**b**) sensitivity analysis

Ten studies compared the hospitalization of Mpox between HIV-positive and HIV-negative patients. Of them, seven studies were included in this analysis. Across the seven studies, the percentage of patients with HIV who had a CD4 count of less than 200 cells/µL was less than 0.5%, as shown in Table 1. The pooled OR was 1.03 (95% CI, 0.77 to 1.36, *P* = 0.85), indicating a comparable hospitalization risk, as shown in Fig. 2a. The studies showed homogeneity, as indicated by an I^2^ value of zero. The other three excluded studies had a high proportion of low CD4 count level in the HIV-positive arm, as indicated by a high RNA viral load or a low CD4 count. Brosnan et al. [30]. found that 51% of the patients had unsuppressed viral loads, and 14% had a CD4 count below 200 cells/µL. Estevez et al. [28]. reported that 59.3% of patients with HIV had a CD4 count below 350 cells/µL, and 9.1% had acquired immune deficiency syndrome (AIDS). Chastain et al. [29] reported that a significant portion of HIV patients had CD4 counts lower than 350 cells/µL. Overall, the risk of hospitalization in these three studies was significantly higher than in HIV-negative patients, with a pooled OR of 2.5 (95% CI, 1.21 to 5.18, *P* = 0.013), as shown in Fig. S1a. An I^2^ value of 0% indicated the studies’ homogeneity.

### Hospitalization risk in HIV-positive patients stratified by CD4 count or HIV RNA load

**Fig. 3:**
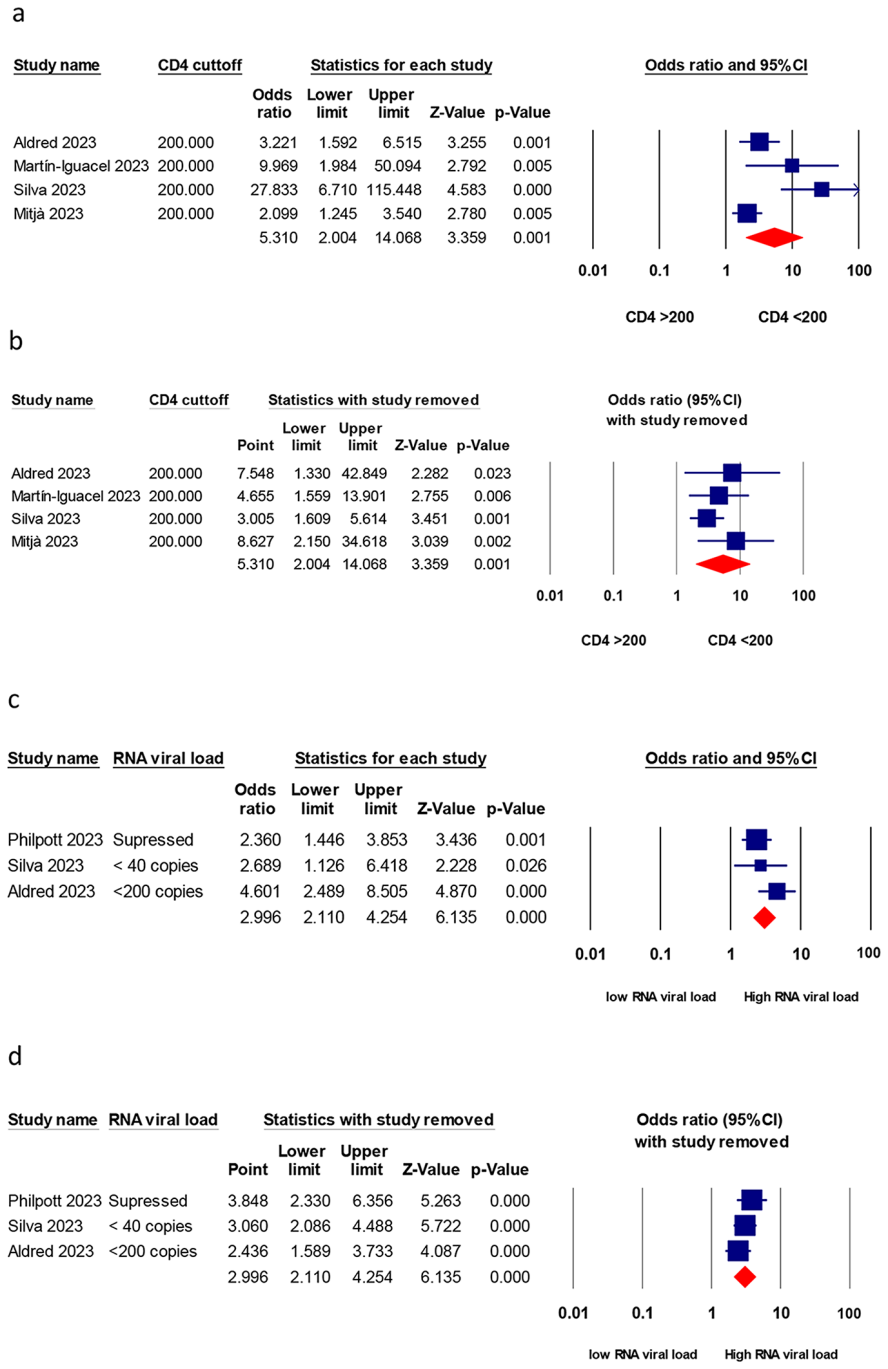
Hospitalization risk in uncontrolled HIV-positive patients compared to controlled HIV-positive patients. (**a**) HIV-positive patients with a low CD4 count (less than 350 cells/µL) compared to high CD4 counts; (**b**) sensitivity analysis. (**c**) HIV-positive patients with an unsuppressed RNA viral load (more than 200 copies/ml) compared to low RNA viral load; (**d**) sensitivity analysis

Five studies reported hospitalization risk stratified by CD4 count, or HIV RNA load as shown in Table 2. Overall, patients with low CD4 counts had a significantly higher hospitalization risk compared to those with higher CD4 counts (> 200 cells/µL), with a pooled OR of 5.3 (95% CI, 2 to 14.06, *P* < 0.001), as depicted in Fig. 3a. An I^2^ value of 77.5% indicated that there was a high heterogeneity among the studies. Similarly, patients with an RNA viral load higher than 200 copies/ml had a significantly higher hospitalization risk compared to those with a lower than 200 copies/ml or undetectable RNA viral load, with a pooled OR of 3 (95% CI, 2.1 to 4.2), as shown in Fig. 3c. An I^2^ value of 0% indicated that the studies were homogeneous.

### The impact of non-HIV immunocompromised condition on Mpox-related hospitalization

As shown in Table 2, Only one report found that cases who were HIV-negative and immunocompromised (84 cases) exhibited a significantly higher risk of hospitalization (adjusted OR = 3.47, 95% CI [1.84 to 6.54], *P* < 0.001) compared to cases who were HIV-negative and immunocompetent [44].

### Mortality in Mpox cases

Among the included studies, 24 reported death events among Mpox cases, with data about immunosuppression sources as shown in Table 3. Most of the reported deaths were observed in patients with HIV with CD4 counts below 200 cells/µL. Seven fatal cases occurred in non-HIV immunosuppressed patients: a case for undiagnosed diabetes [16], five cases for kidney transplant recipients [16, 39, 43, 45] and a case for a patient with allogeneic stem cell transplantation [32]. The main severe manifestations that could contribute to mortality were severe sepsis from secondary bacterial infections, respiratory complications such as pulmonary nodules and airway occlusion, and gastrointestinal complications such as severe proctitis, intestinal obstruction, and bleeding rectal ulcers, as shown in Table S5.

### The mechanisms contributing to the severity of Mpox in immunocompromised individuals

Mpox has the potential to cause severe infection or even death through three main mechanisms. The first involves the dissemination of the virus through the bloodstream or lymph system, leading to direct organ damage as confirmed by autopsy and biopsy findings [11, 13, 37, 39, 41–43]. The second mechanism is the initiation of immune dysregulation, which can manifest as conditions like hemophagocytic lymphohistiocytosis (HLH) [11–13] or immune reconstitution inflammatory syndrome (IRIS) [12, 14–19]. Lastly, Mpox can also contribute to the development of severe sepsis because of secondary bacterial infections as shown in Table S5.

### Evidence of disseminating Mpox based on autopsy findings of death cases

Based on the autopsy findings, Mpox was confirmed through PCR or immunostaining tests in multiple organs (Table S6). These organs, including the skin, digestive tract, lungs, liver, spleen, lymph nodes, kidneys, testes, heart, brain, bone marrow, tongue, salivary gland, and adrenal gland [11, 13, 37, 39, 41–43]. The virus was also detected in circulating white blood cells [39, 43].

### The potential role of immune dysregulation in clinical deterioration and death

There were three reported cases suspected of HLH [11–13]. In one case, Govind et al. suspected HLH based on clinical features such as profound leukocytosis, septic shock physiology, and abnormalities like anemia, thrombocytopenia, elevated liver enzymes, ferritin, and soluble CD25 [12]. Alarcón et al. reported a case diagnosed with HLH based on bone marrow histology [11]. Additionally, based on autopsy findings, Duarte-Neto et al. found that Mpox triggers macrophage dysfunction with hemophagocytosis, raising concerns for HLH [13]. On the other hand, seven studies with data on the timing of initiating or reinitiating antiretroviral therapies (ART) suspected IRIS. In Mitjà et al.‘s study, 21 out of 85 people who initiated or restarted ART were suspected of having IRIS [15]. Riser et al. suspected IRIS in 13 out of 19 individuals receiving ART [16]. The five other studies showed that all deaths reported were suspected to have IRIS [12, 14, 17–19]. Of those studies, one documented immune recovery through a decline in HIV viral load and an increase in CD4 cell count following ART initiation [12].

### The evidence of concomitant non-mpox infections at the time of hospitalization and death

Most of the included reports show concomitant non-Mpox infections at the time of hospitalization and death, as shown in Table 3. A comprehensive list of the non-MPOX pathogens for each study is shown in Tables S5. Overall, a diverse range of pathogens were identified, including viral agents such as BK virus, CMV, adenovirus, human parainfluenza virus-3, and Epstein-Barr virus, both gram-positive and gram-negative bacteria, fungal infections, and HIV-associated opportunistic infections, such as Pneumocystis jiroveci pneumonia, visceral leishmaniasis, Kaposi sarcoma, CMV retinitis, disseminated TB, and esophageal candidiasis.

### Sensitivity analysis

The sensitivity analyses by leave-one-out revealed that all pooled estimates are reliable and do not depend on any study removed, as shown in Figs. 2b and 3b and d, S1b, and S2.

## Discussion

In this systematic review and meta-analysis, we assessed the impact of HIV or non-HIV-associated immunosuppression on the severity of Mpox requiring hospitalization and reviewed all fatality cases in the current 2022 outbreak. Our findings demonstrate that people with HIV with CD4 cell counts > 200 cells/µL do not worsen Mpox hospitalization compared to HIV-negative individuals. Yet, individuals with HIV with CD4 cell counts < 200 cells/µL or RNA VL > 200 copies/ml increase hospitalization risk compared to controlled HIV-positive patients. Most of the fatal cases occurred in patients with HIV CD4 cell counts less than 200 cells/µL, with some cases in non-HIV immunocompromised patients. These findings show that Mpox acts as an opportunistic pathogen in immunocompromised individuals and underscore the importance of close monitoring for all immunocompromised patients experiencing Mpox, regardless of HIV status.

Our study revealed that individuals with HIV and CD4 cell counts greater than 200 cells/µL did not experience an increased risk of Mpox-related hospitalization compared to HIV-negative individuals. The pooled odds ratio was 1.03 (95% CI, 0.77 to 1.36, *P* = 0.85), indicating no significant difference between the two groups. These findings are supported by a global surveillance analysis conducted by WHO Member States, which reported that non-immunosuppressed individuals with HIV did not exhibit higher odds of hospitalization (adjusted OR = 0.91, 95% CI [0.71 to 1.16], *P* = 0.44). However, this study lacked data on the CD4 count or RNA viral load of HIV patients, and there was no specific definition provided for what constitutes immunosuppression [44].

To the best of our knowledge, this is the first comprehensive systematic review with a meta-analysis that investigates the impact of HIV on Mpox-related hospitalization and mortality. However, there are several meta-analyses on the impact of HIV on hospitalization and mortality associated with COVID-19.

These meta-analyses yielded inconsistent findings, with three of them indicating an association between HIV and an elevated risk of mortality and hospitalization [47–49], while one study found no such association [50]. A common limitation across all these studies is the absence of subgroup analyses considering RNA viral load and CD4 levels. According to our findings, Patients with a CD4 count lower than 200 cells/µL or an unsuppressed RNA viral load (> 200 copies/ml) had a significantly higher hospitalization risk (OR = 5.3, *P* < 0.001) and (OR = 3, *P* < 0.001), respectively. This suggests that RNA viral load and CD4 levels significantly influence Mpox-related hospitalization and mortality and underscores the crucial need for future research to incorporate stratification based on these key immune markers for a more precise estimation of disease severity.

Twenty-four studies reported death events among Mpox cases with data about immunosuppression sources. Most fatal events were observed in HIV-positive individuals with CD4 counts below 200 cells/µL. Seven fatal cases occurred in non-HIV immunosuppressed patients: a case for undiagnosed diabetes [16], five cases for kidney transplant recipients [16, 39, 43, 45], and a case for a patient with allogeneic stem cell transplantation [32]. These findings suggest the importance of expanding treatment considerations for individuals with immunosuppression, regardless of their HIV status. Similarly, the CDC expanded interim clinical treatment considerations for severe manifestations of Mpox to include people with immunocompromising conditions other than HIV [51]. The basis for this recommendation stems from a report highlighting severe manifestations in patients with immunocompromising conditions caused by non-Mpox viruses belonging to the same family, including smallpox, molluscum contagiosum, and orf virus [52], and a fatal case of cowpox virus infection previously reported in a recipient of a kidney transplant [53] to infer potential complications that may apply to Mpox. The CDC also mentioned that it is currently uncertain whether the evidence can be generalized to Mpox [51]. However, fatal cases of the current 2022 Mpox outbreak were documented in non-HIV immunosuppressed individuals [16, 32, 39, 43, 45], providing support for the special risk of severe manifestation of Mpox in those individuals.

Mpox primarily enters the human body through direct contact with infectious skin lesions, respiratory droplets, or mucosal surfaces. Once inside, the virus targets and replicates within epithelial cells at the initial entry site [54]. Following the incubation period, the virus spreads from the point of entry through the bloodstream or lymphatic system to infect distant organs. The detection of Mpox in circulating leukocytes and lymph nodes suggests these routes of dissemination [39, 43]. The autopsy findings revealed the presence of the virus in various organs and tissues such as the skin, digestive tract, lungs, liver, spleen, lymph nodes, kidneys, testes, heart, brain, bone marrow, tongue, salivary gland, and adrenal gland [11, 13, 37, 39, 41–43]. The virus was also detected in circulating white blood cells [39, 43]. The broad range of organ involvement observed in Mpox infection is in line with findings from a previous study conducted on mice, indicating a consistent pattern of wide tissue tropism [55]. In individuals with severe immunocompromise, widespread Mpox infection can lead to significant tissue damage and organ failure through three mechanisms. Three primary methods contribute to the severity and even death of Mpox cases. The first involves the dissemination of the virus through the bloodstream or lymph, leading to direct organ damage. The second is that it can contribute to the development of severe sepsis because of secondary bacterial infections, as presented in Table S5. Lastly, it can initiate immune dysregulation, which can manifest as conditions like HLH [11–13] or IRIS [12, 14–19]. IRIS lacks a widely accepted definition. However, the diagnosis of IRIS requires an acute worsening of the condition after initiating or reinitiating ART in patients with low CD4 counts (often less than 100 cells/µL) with evidence of immunological recovery [56]. Seven studies with data about the clinical deterioration of Mpox after initiating or reinitiating ART suspected IRIS [14–16, 18, 19, 32]. Of the seven studies, one documented immune recovery through a decline in HIV viral load and an increase in CD4 cell count following ART initiation [12]. Therefore, it is questionable if these cases represent IRIS, the progression of Mpox, or the progression of undiagnosed opportunistic infections. Additionally, three fatal cases were suspected of HLH [11–13]. HLH is characterized by immune hyperresponse and excessive inflammation, resulting in significant tissue damage [57]. The underlying cause of this excessive inflammation is often attributed to the failure of normal downregulation mechanisms in activated macrophages and lymphocytes [57]. In patients with HLH, there is persistent activation of macrophages, natural killer cells, and cytotoxic T lymphocytes, leading to the overproduction of cytokines (known as cytokine storms) by these cells [57, 58]. Excessive cytokine levels are believed to be responsible for the development of multiorgan failure [57, 58]. Further research is crucial to improving our understanding of the mechanism behind Mpox severity.

Mitjà et al. proposed designating fulminant or necrotizing Mpox as an AIDS-defining condition [59]. However, Núñez et al. do not advocate for this labeling for two key reasons [60]. Firstly, it could unwarrantedly stigmatize individuals. Secondly, it risks misleading physicians about the exclusive relationship between advanced HIV and severe Mpox. However, our study underscored six fatal cases of Mpox in non-HIV immunosuppressed individuals [16, 32, 39, 43]. This suggests that a severe form of Mpox is not exclusive to patients with advanced HIV and can occur in individuals with other forms of immunosuppression as well. Designating a severe form of Mpox as an AIDS-defining condition may be misguided. We agree with Núñez et al. [61]. that a more suitable label would be opportunistic infection. This classification would cue clinicians to consider the disease as potentially more serious in anyone who is immunosuppressed, not just those with HIV/AIDS.

This study has implications for both clinical practice and research. Our findings suggest that individuals with uncontrolled HIV (CD4 count is less than 200 cells/µL or RNA viral load higher than 200 copies/ml) or with non-HIV immunocompromising conditions had poor Mpox outcomes and should be prioritized for vaccination, early treatment, and monitoring for worrying clinical outcomes. Future research on the impact of HIV on Mpox should stratify their outcomes by CD4 count and viral load, as these factors may influence the severity and progression of the disease. Researchers should also adjust for potential confounding variables, such as other non-HIV immunocompromised conditions, as these conditions are common in people with advanced HIV and could cause the results to be overstated. Finally, more research on immunopathogenesis in severe MPox patients is required to investigate the possible role of immunological dysregulation, in particular IRIS or HLH, in clinical decline and death.

### Limitation

One limitation of our findings is the absence of a comparative study on mortality, likely due to the low overall mortality rate observed in the current outbreak. Additionally, the evidence supporting poor outcomes in individuals with non-HIV immunosuppression is currently limited to a few studies.

## Conclusion

This study demonstrates that people with HIV with a CD4 count of less than 200 cells/µL or an unsuppressed viral load have a higher risk of severe Mpox requiring hospitalization. Furthermore, most fatal cases were observed in HIV patients with a CD4 count of less than 200 cells/µL, with some cases in non-HIV immunocompromised patients, indicating that severe outcomes are not limited to advanced HIV patients alone. These findings show that Mpox acts as an opportunistic pathogen in immunocompromised individuals. Therefore, it is imperative to prioritize early care and closely monitor the health of these patients to detect any deterioration.

### Electronic supplementary material

Below is the link to the electronic supplementary material.

Supplementary Material 1

## Data Availability

All data generated and analyzed throughout this study were included either in this article or its supplementary information file.

## Abbreviations

MPXV: Monkeypox
HIV: Human Immunodeficiency Virus
OR: Odds Ratio
HLH: Hemophagocytic Lymphohistiocytosis
IRIS: immune reconstitution inflammatory syndrome
WHO: World Health Organization
PHEIC: Public Health Emergency of International Concern
FDA: Food and Drug Administration
NOS: The Newcastle-Ottawa Scale
CDC: The Centers for Disease Control and Prevention
AIDS: Acquired Immunodeficiency Syndrome
PRISMA: Preferred Reporting Items for Systematic Reviews and Meta-Analyses
CI: Confidence Interval
ART: Antiretroviral Therapy
CMV: Cytomegalovirus
VL: Viral Load
RR: Risk Ratio
PWoH: People without HIV
PWH: People with HIV
ARDS: Acute Respiratory Distress Syndrome
