# Supplementary material for: The impact of immunosuppression on the mortality and hospitalization of Monkeypox: a systematic review and meta-analysis of the 2022 outbreak
Source: Virol J. 2024 Jun 5;21:130. doi: 10.1186/s12985-024-02392-0 (PMC11155170; doi:10.1186/s12985-024-02392-0)
Supplement: Supplementary file 1 — Supplementary Material 1 [file 12985_2024_2392_MOESM1_ESM.docx]

**Supplementary file**

**The Impact of Immunosuppression on the Mortality and Hospitalization of Monkeypox: A Systematic Review and Meta-Analysis of the 2022 Outbreak**

Ahmed Azzam^1^*, Heba Khaled^2^, Haitham Salem^3^, Ameer Ahmed^4^, Amira M. Heniedy^5^, Hassan Samy Hassan^6^, Ahmed Hassan^7^, Taghrid S. El-Mahdy^1, 8^

^1^ Department of Microbiology and Immunology, Faculty of Pharmacy, Helwan University, Cairo, Egypt.
^2^ Department of Biochemistry, Faculty of Pharmacy, Cairo University, Cairo, Egypt.
^3^ Faculty of Medicine Ain Shams University, Cairo, Egypt.
^4^ Faculty of Medicine, Minia University, Minya, Egypt.
^5^ Department of epidemiology, Elbuhyra veterinary administration, Egyptian ministry of agriculture, Elbuhyra, Egypt.
^6^ Faculty of Pharmacy, Tanta University, Tanta, Egypt.
^7^ Dermatology resident physician, Qeft Teaching Hospital, Qena, Egypt
^8^ Department of Microbiology and Immunology, Faculty of Pharmacy, Modern University for Technology and Information (MTI), Cairo, Egypt.

***Corresponding author: Ahmed Azzam**
**Department of Microbiology and Immunology, Faculty of Pharmacy, Helwan University, Cairo Egypt.**

**Telephone: ‎+20 0237222210**

**Email: ahmed.abdelkareem@pharm.helwan.edu.eg**

**Table S1: The search strategy used in this study**

| String | Search Terms |
| --- | --- |
| #1 | monkeypox OR “monkey pox” OR mpox OR MPXV |
| #2 | “Human immunodeficiency virus” OR "acquired immunodeficiency syndrome" OR AIDS OR HIV OR HIV-1 OR HIV-2 OR immunocompromis* OR immunosuppress*, OR "immune suppression", OR "immune deficiency", "weakened immune system", OR "compromised immunity", OR immunodeficiency, OR chemotherapy, OR transplant*, OR malignan* |
| #3 | hospitaliz* OR death OR mortality OR die* OR fatal |
| #4 | #1 AND #2 AND #3 AND #4 |

**Table S2:** Supplementary preferred reporting items for systematic reviews and meta-analyses (PRISMA) checklist.

| **Section/topic** | **Item No** | **Checklist item** | **Reported on page No** |
| --- | --- | --- | --- |
| **Title** | 1 | Identify the report as a systematic review, meta-analysis, or both | 1 |
| **Abstract** | | | |
| **Structured summary** | 2 | Provide a structured summary including, if applicable, background, objectives, data sources, study eligibility criteria, participants, interventions, study appraisal and synthesis methods, results, limitations, conclusions and implications of key findings, systematic review registration number | 2 |
| **Introduction** | | | |
| **Rationale** | 3 | Describe the rationale for the review in the context of what is already known | 3, 4 |
| **Objectives** | 4 | Provide an explicit statement of questions being addressed with reference to participants, interventions, comparisons, outcomes, and study design (PICOS) | 3, 4 |
| **Methods** | | | |
| **Protocol and registration** | 5 | Indicate if a review protocol exists, if and where it can be accessed (such as web address), and, if available, provide registration information including registration number | - |
| **Eligibility criteria** | 6 | Specify study characteristics (such as PICOS, length of follow-up) and report characteristics (such as years considered, language, publication status) used as criteria for eligibility, giving rationale | 4 |
| **Information sources** | 7 | Describe all information sources (such as databases with dates of coverage, contact with study authors to identify additional studies) in the search and date last searched | 5 |
| **Search** | 8 | Present a full electronic search strategy for at least one database, including any limits used, such that it could be repeated | Table S1 |
| **Study selection** | 9 | State the process for selecting studies (that is, screening, eligibility, included in the systematic review, and, if applicable, included in the meta-analysis) | 5 |
| **Data collection process** | 10 | Describe the method of data extraction from reports (such as piloted forms, independently, in duplicate) and any processes for obtaining and confirming data from investigators | 5 |
| **Data items** | 11 | List and define all variables for which data were sought (such as PICOS, and funding sources) and any assumptions and simplifications made | 5 |
| **Risk of bias in individual studies** | 12 | Describe methods used for assessing the risk of bias in individual studies (including specification of whether this was done at the study or outcome level), and how this information is to be used in any data synthesis | **Tables S4** |
| **Summary measures** | 13 | State the principal summary measures (such as risk ratio, and difference in means). | 5, 6 |
| **Synthesis of results** | 14 | Describe the methods of handling data and combining results of studies, if done, including measures of consistency (such as I^2^ statistic) for each meta-analysis | 5, 6 |
| **Risk of bias across studies** | 15 | Specify any assessment of risk of bias that may affect the cumulative evidence (such as publication bias, selective reporting within studies) | 5, 6 |
| **Additional analyses** | 16 | Describe methods of additional analyses (such as sensitivity or subgroup analyses, meta-regression), if done, indicating which were pre-specified | 5, 6 |
| **Results** | | | |
| **Study selection** | 17 | Give numbers of studies screened, assessed for eligibility, and included in the review, with reasons for exclusions at each stage, ideally with a flow diagram | Fig. 1 |
| **Study characteristics** | 18 | For each study, present characteristics for which data were extracted (such as study size, PICOS, follow-up period) and provide the citations | Tables 1-3 |
| **Risk of bias within studies** | 19 | Present data on the risk of bias of each study and, if available, any outcome-level assessment (see item 12). | **Table S4** |
| **Results of individual studies** | 20 | For all outcomes considered (benefits or harms), present for each study (a) simple summary data for each intervention group and (b) effect estimates and confidence intervals, ideally with a forest plot | Figs. 2 and 3 and pages 6 and 8 |
| **Synthesis of results** | 21 | Present results of each meta-analysis done, including confidence intervals and measures of consistency | Figs. 2 and 3  and pages 6 and 8 |
| **Risk of bias across studies** | 22 | Present results of any assessment of the risk of bias across studies (see item 15) | - |
| **Additional analysis** | 23 | Give results of additional analyses, if done (such as sensitivity or subgroup analyses, meta-regression) (see item 16) | Figs. 2b, 3b, 3d, S1b, and S2. |
| **Discussion** | | | |
| **Summary of evidence** | 24 | Summarize the main findings including the strength of evidence for each main outcome; consider their relevance to key groups (such as health care providers, users, and policymakers) | 13 |
| **Limitations** | 25 | Discuss limitations at the study and outcome level (such as the risk of bias), and at the review level (such as incomplete retrieval of identified research, reporting bias) | 17 |
| **Conclusions** | 26 | Provide a general interpretation of the results in the context of other evidence, and implications for future research | 17 |
| **Funding** | | | |
| **Funding** | 27 | Describe sources of funding for the systematic review and other support (such as the supply of data) and the role of funders for the systematic review | 18 |

**Table S3: NEWCASTLE - OTTAWA QUALITY ASSESSMENT SCALE** **COHORT STUDIES**

**Selection**

1) Representativeness of the exposed cohort

a) truly representative of the average _______________ (describe) in the community **🟑**

b) somewhat representative of the average ______________ in the community **🟑**

c) selected group of users eg nurses, volunteers

d) no description of the derivation of the cohort

2) Selection of the non-exposed cohort

a) drawn from the same community as the exposed cohort **🟑**

b) drawn from a different source

c) no description of the derivation of the non-exposed cohort

3) Ascertainment of exposure

a) secure records (eg surgical records) **🟑**

b) structured interview **🟑**

c) written self-report

d) no description

4) Demonstration that outcome of interest was not present at start of study

a) yes **🟑**

b) no

**Comparability**

5) Comparability of cohorts based on the design or analysis

a) study controls for _____________ (select the most important factor) **🟑**

b) study controls for any additional factor **🟑** (These criteria could be modified to indicate specific control for a second important factor.)

**Outcome**

6) Assessment of outcome

a) independent blind assessment **🟑**

b) record linkage **🟑**

c) self-report

d) no description

7) Was follow-up long enough for outcomes to occur

a) yes (select an adequate follow-up period for the outcome of interest) **🟑**

b) no

8) Adequacy of follow-up of cohorts

a) complete follow-up - all subjects accounted for **🟑**

b) subjects lost to follow-up unlikely to introduce bias - small number lost - > ____ % (select an adequate %) follow up, or description provided of those lost) **🟑**

c) follow up rate < ____% (select an adequate %) and no description of those lost

d) no statement

| **Study** | **Selection** | | | | **Comparability** | **Outcome** | | | **Total (9/9)** |
| --- | --- | --- | --- | --- | --- | --- | --- | --- | --- |
|  | Q1^β^ | Q2 | Q3 | Q4 | Q5 | Q6 | Q7 | Q8 |  |
| Angelo 2023 | * | * | * | * | * | * | * | * | 8 |
| Caria 2022 | * | * | * | * | * | * | * | * | 8 |
| Hoffmann 2023 | * | * | * | * | * | * | * | * | 8 |
| Pilkington 2023 | * | * | * | * | * | * | * | * | 8 |
| Brosnan 2023 | * | * | * | * | 0 | * | * | * | 7 |
| Estevez 2023 | * | * | * | * | 0 | * | * | * | 7 |
| Chastain 2023 | * | * | * | * | 0 | * | * | * | 7 |
| Philpott, 2023 | * | * | * | * | * | * | * | * | 8 |
| Martín-Iguacel, 2023 | * | * | * | * | * | * | * | * | 8 |
| Mitjà, 2023[30] | * | * | * | * | * | * | * | * | 8 |
| Silva, 2023 | * | * | * | * | * | * | * | * | 8 |
| Aldred, 2023 | * | * | * | * | * | * | * | * | 8 |
| Laurenson-Schafer, 2023 | * | * | * | * | * | * | * | * | 8 |

**Table S4: NEWCASTLE - OTTAWA quality assessment scale** **cohort studies**

Note: β the checklist items are presented in table S2

**Table S5: Primary contributing factor to death and concomitant non-Mpox infection at the time of hospitalization and death**

| Author | Country | # death, HIV status | Primary contributing  factor to death | Non-Mpox concomitant  infections |
| --- | --- | --- | --- | --- |
| Garneau, 2023 | USA | 2 (+) | Diffuse necrotic wounds needing debridement, complicated by bacteremia and multisystem organ failure | - |
| Aldred, 2023 | USA | 1 (+) | NA | - |
| Mitjà, 2023 | Multinational | 27 (+) | Of the total 27 deaths:20 septic shock and multiorgan failure; 4 were respiratory failures; 2 were disseminated Mpox; and 1 had cardiac arrest. | HIV-associated opportunistic infections: Pneumocystis jiroveci Pneumonia, Visceral leishmaniasis, Kaposi sarcoma, CMV retinitis, disseminated TB, esophageal Candidiasis  ESBL E. coli bacteremia, Pseudomonas aeruginosa bacteremia, Clostridium sporogenes bacteremia, K. pneumoniae skin infection, P. aeruginosa skin infection, E. faecalis skin infection |
| Silva, 2023 | Brazil | 2 | Bowel and urologic obstruction, sepsis, and refractory shock | - |
| Núñez, 2022 | Mexico | 1 | Septic shock and respiratory failure | hepatitis C, and syphilis |
| Riser, 2023 | USA | (33 (+) available clinical data were missing) | - | - |
| Álvarez-Moreno, 2023 | Colombia | 2 (+) | Fatal septic shock in the first fetal case and necrotizing fasciitis in the other case. | yes |
| Caria, 2023 | Brazil | 1(+) | Septic shock  (The patient's sepsis was caused by a hospital-acquired *Acinetobacter baumannii*) | Acinetobacter baumannii pneumonia |
| Carrubba, 2023 |  | 2 (+) | For the first case (extensive facial necrosis and eye damage, bleeding rectal ulcers led to hemorrhagic shock, ulcerations in various organs, and secondary infections). For the second case (extensive skin necrosis and multi-organ failure, unstable airway requiring tracheostomy, and multifocal pneumonia). | Sapovirus gastroenteritis |
| Menezes, 2022[ | Brazil | 1(+) | Severe sepsis associated with multiple organ dysfunctions | - |
| Farias, 2023 | Brazil | 1(+) | The patient developed intestinal obstruction, possibly related to Mpox, which necessitated surgery. Post-surgery, he developed intra-abdominal bacterial sepsis that ultimately led to death due to refractory septic shock. | Enterococcus faecium vancomycin-resistant bacteria |
| Rajme-López, 2023 | Mexico | 3(+) | Overall, the cause of death in the three cases were determined to be Mpox-related multiple organ failure since co-infections and opportunistic pathogens were excluded as contributing factors. Two cases necessitated a tracheostomy procedure due to airway occlusion; one patient experienced complete airway blockage, leading to severe hypoxemia and subsequent cardiac arrest and death. lung nodules were identified in two cases. | No |
| Warner, 2023 | USA | 1(+) | Extensive and worsening skin lesions and multi-organ failure (acute encephalopathy, acidosis, and acute kidney injury). | cryptococcal meningitis |
| Triana-González, 2023 | Mexico | 5 (4+) and (1-) | The first case: (soft tissue infection, intraabdominal infection, and intestinal obstruction), the second case: (intestinal obstruction, bacterial infections (intraabdominal infection, pneumonia), ARDS and bilateral pleural effusion and multiple pulmonary nodules), the third case: (perinatal abscess, rectal prolapse, proctitis, disseminated inflammatory lymph nodes, hepatosplenomegaly, bacterial infections, and ARDS), the fourth case (ARDS and Gram-negative bacteremia) and the fifth case: (ARDS due to bilateral pleural effusion and multiple pulmonary nodules, recurrent angioedema due to disseminated histoplasmosis) | ESBL E. coli, Enterococcus faecium, Pseudomonas aeruginosa, carbapenem-resistant Acinetobacter baumannii, methicillin-susceptible Staphylococcus aureus |
| Petti, 2023 | USA | 2 (+) | Multi-organ failure | CMV viremia, severe/fulminant Clostridioides difficile infection, Pseudomonas aeruginosa bacteremia, ESBL E. coli bacteremia, Enterococcus gallinarum bacteremia, adenovirus |
| Filippov, 2023 | USA | 1(+) | Extensive and worsening skin lesions, several lung nodules and a large left-sided pleural effusion led to severe respiratory distress and the death of cardiac arrest. | - |
| Alarcón 2023 | USA | 1(+) | Mpox-related severe proctitis likely initiated the severe sepsis associated with septic shock and renal failure. | Staphylococcus epidermidis bacteremia |
| Fuller 2023 | USA | 1(-) | The mpox virus disseminated throughout the body, causing severe complications such as encephalopathy, respiratory failure, shock, multiorgan failure, and acute liver failure. | BK virus, cytomegalovirus (CMV) |
| Govind 2023[ | USA | 3(+) | Case 1: severe proctitis, facial necrotic lesions, massive rectal bleeding, hemorrhagic shock, acute liver injury, secondary bacterial infection, and candidemia. Case 2: severe proctitis, necrotic lesions, rectal pain and bleeding, respiratory failure, septic shock, abdominal compartment syndrome, tension pneumothorax, ischemic hepatitis, and cardiac arrest.  Case 3: facial necrotic lesions, bowel obstruction, bilateral pleural effusions, airway occlusion, renal failure, respiratory failure, and refractory hypotension. | CMV infection, Enterobacter cloacae bacteremia, Klebsiella pneumoniae bacteremia, antifungal-resistant Candida parapsilosis |
| Ritter, 2024 | USA | 22, 20 (HIV +), 2(HIV -) | Necrotic lesions in the digestive tract, the lungs, lymphoid tissues, and several other organs. | Various non-Mpox coinfections: CMV, Candida spp., non-pneumococcal Streptococcus spp., human parainfluenza virus-3, Pneumocystis spp., mixed bacteria (Escherichia coli and Staphylococcus spp.) |
| Duarte-Neto, 2023 | Brazil | 2 (+) | Case 1: extensive necrotic ulcers on the airway tracts had obstructed the bronchial ostia of the upper segments of both lower lobes, severe hypoxia, cardiopulmonary arrest, and nosocomial septic shock.  Case 2: intestinal obstruction, exploratory laparotomy-detected diffuse colitis with edema of the colonic walls, and airway obstruction with cardiac arrest. | Case1: Influenza A and B, Acinetobacter baumannii pneumonia and tuberculosis and histoplasmosis  Case2: T pallidum infections |
| Mohammed, 2023 | USA | 1(+) | Acute liver injury, lesions in the esophagus and stomach, respiratory failure, and sepsis. | CMV, BK, and EBV |
| SUN, 2023 | - | 1(+) | Bilateral lung nodules, necrotizing pneumonia, Sepsis | Pseudomonas Bacteremia |
| Higgins, 2023 | USA | 1(-) | Pneumonitis and shock patient. | CMV reactivation |

**Table S6: Autopsy findings of deaths among Mpox cases**

| Viral detection methods | Organ | Specimen | Author |
| --- | --- | --- | --- |
| immunohistochemistry (IHC) and in situ hybridization (ISH) | Skin, Digestive tract, Lung, Lymphoid tissues, Brain, Liver Adrenal gland, Testis, Pancreas and circulating leukocytes | autopsy | Ritter 2024 |
| immunostaining | skin, esophagus, lungs, liver, spleen, lymph nodes, kidneys, and testis; in circulating leukocytes in heart and brain. | autopsy | Fuller 2023 |
| PCR | Skin, brain, bone marrow, and testicles. | autopsy | Alarcón 2023 |
| Detectable vaccinia antigens and PCR | Skin, lungs, digestive tract, tongue, pancreas, Salivary glands, testes, adrenal glands, kidneys, heart and liver. | autopsy | Duarte-Neto 2023 |
| - | esophagus and stomach. | autopsy | Mohamed 2023 |
| - | Lung | autopsy | Sun 2023 |
| - | multiple organs | autopsy | Carrubba 2023 |

**a**

**b**

**Figure S1: The pooled hospitalization risk among HIV-positive patients with a high proportion of CD4 counts of less than 350 cells/μL in HIV-positive patients compared with HIV-negative control. (a) the pooled analysis; b) the sensitivity analysis.**
